# Identification and Characterization of Quorum-Quenching Activity of *N*-Acylhomoserine Lactonase from Coagulase-Negative Staphylococci

**DOI:** 10.3390/antibiotics9080483

**Published:** 2020-08-05

**Authors:** Tomohiro Morohoshi, Yaoki Kamimura, Nobutaka Someya

**Affiliations:** 1 Department of Material and Environmental Chemistry, Graduate School of Engineering, Utsunomiya University, Utsunomiya 321-8585, Japan; yaokami@gmail.com; 2 Institute of Vegetable and Floriculture Science, National Agriculture and Food Research Organization, 3-1-1 Kannondai, Ibaraki 305-8519, Japan; someyan@affrc.go.jp

**Keywords:** quorum sensing, *N*-acylhomoserine lactone, coagulase-negative staphylococci, AHL lactonase, *Pseudomonas aeruginosa*

## Abstract

*N*-Acylhomoserine lactones (AHLs) are used as quorum-sensing signals in Gram-negative bacteria. Many genes encoding AHL-degrading enzymes have been cloned and characterized in various microorganisms. Coagulase-negative staphylococci (CNS) are present on the skin of animals and are considered low-virulent species. The AHL-lactonase gene homologue, *ahlS*, was present in the genomes of the CNS strains *Staphylococcus carnosus*, *Staphylococcus haemolyticus*, *Staphylococcus saprophyticus*, and *Staphylococcus sciuri*. We cloned the candidate *ahlS* homologue from six CNS strains into the pBBR1MCS5 vector. AhlS from the CNS strains showed a higher degrading activity against AHLs with short acyl chains compared to those with long acyl chains. AhlS from *S. sciuri* was expressed and purified as a maltose-binding protein (MBP) fusion. *Pseudomonas aeruginosa* is an opportunistic pathogen that regulates several virulence factors such as elastase and pyocyanin by quorum-sensing systems. When MBP-AhlS was added to the culture of *P. aeruginosa* PAO1, pyocyanin production and elastase activity were substantially reduced compared to those in untreated PAO1. These results demonstrate that the AHL-degrading activity of AhlS from the CNS strains can inhibit quorum sensing in *P. aeruginosa* PAO1.

## 1. Introduction

Quorum sensing is a cell-to-cell communication system used by many bacterial species depending on their population densities [1]. One of the most common quorum-sensing signals, *N*-acyl-l-homoserine lactone (AHL), is used by Gram-negative bacteria [2]. Once the AHL reaches a threshold concentration, the transcription of specific genes is activated, resulting in the expression of phenotypes such as motility, adhesion, biofilm formation, toxicity, and pathogenicity [3]. AHL-negative mutants of many pathogens generally have defected pathogenicity [3,4]. Quorum quenching, which disrupts or manipulates quorum-sensing signals, is one of the most effective techniques to inhibit the expression of virulence and disrupt the infection of host cells. Many AHL-degrading enzymes have been cloned from various microorganisms and studied for their applications in the control of infectious diseases [5]. AHL-degrading enzymes have been divided into two functional groups—AHL lactonase and AHL acylase [5]. AHL lactonase catalyzes AHL ring-opening by hydrolyzing lactones, whereas AHL-acylase hydrolyzes the amide bond of AHL [5]. AiiA-type AHL lactonase, first identified from *Bacillus* sp. 240B1, is the most-studied AHL-degrading enzyme [6]. It belongs to the metallo-β-lactamase superfamily and has been identified and characterized in various bacteria such as AhlS from *Solibacillus silvestris* [7], AttM from *Agrobacterium tumefaciens* [8], AhlD from *Arthrobacter* sp. [9], and AidC from *Chryseobacterium* sp. [10]. In addition, we have previously reported a highly-thermostable AiiA-type AHL lactonase, AiiT, which was isolated from the thermophilic bacterium *Thermaerobacter marianensis* JCM 10246 [11].

Coagulase-negative staphylococci (CNS) are known to comprise over 30 species, which are part of the normal flora on the skin of animals [12]. Coagulase, which converts fibrinogen into fibrin, is a major virulence factor produced by coagulase-positive staphylococci (CPS), e.g., *Staphylococcus aureus* and *Staphylococcus pseudintermedius* [12]. Therefore, CNS strains are considered less virulent than CPS strains. Gram-positive bacteria such as CNS strains do not produce any AHL molecules but use oligopeptide as their quorum-sensing signals [1,3]. Meanwhile, some Gram-positive bacteria produce AHL-degradative enzyme and inhibit quorum sensing in other Gram-negative bacteria [5]. So far, AHL-degradative enzymes isolated from CNS strains have not been reported. In this study, we identified and characterized novel AHL-degradative genes from several CNS strains.

## 2. Results and Discussion

### 2.1. AHL-Degrading Activities in CNS Strains

**Figure 1:**
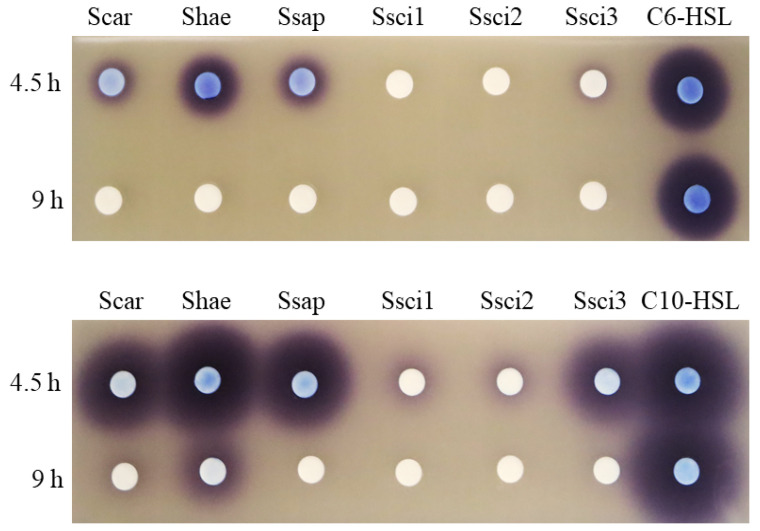
*N*-Acylhomoserine lactone (AHL)-degrading activities of CNS strains. Full-grown cultures of CNS strains, *Staphylococcus carnosus* NBRC 109622 (Scar), *Staphylococcus haemolyticus* NBRC 109768 (Shae), *Staphylococcus saprophyticus* NBRC 102446 (Ssap), *Staphylococcus sciuri* American Type Culture Collection (ATCC) 29060 (Ssci1), *S. sciuri* 29061 (Ssci2), and *S. sciuri* StLB252 (Ssci3) were mixed with 20 μM *N*-hexanoyl-l-homoserine lactone (C6-HSL) or *N*-decanoyl-l-homoserine lactone (C10-HSL) and incubated at 37 °C for 4.5 and 9 h. The residual AHLs were detected using *Chromobacterium violaceum* CV026 (for C6-HSL) or VIR07 (for C10-HSL).

Six strains from four CNS species, *Staphylococcus carnosus*, *Staphylococcus haemolyticus*, *Staphylococcus saprophyticus*, and *Staphylococcus sciuri* were selected to evaluate their AHL-degrading activities. CNS strains were cultivated in Luria–Bertani (LB) liquid medium containing 20 μM *N*-hexanoyl-l-homoserine lactone (C6-HSL) or *N*-decanoyl-l-homoserine lactone (C10-HSL). After 4.5 and 9 h incubation, the remaining AHLs in the culture supernatant were visualized by AHL biosensors, *Chromobacterium violaceum* CV026 (for the short chain C6-HSL) and VIR07 (for the long chain C10-HSL), which produced the purple pigment violacein [13,14]. The results of AHL degradation are shown in Figure 1. All CNS strains used in this study completely degraded 20 μM C6-HSL by 9 h incubation, confirming their C6-HSL-degrading activities. Specifically, *S. sciuri* ATCC 29060 and 29061 completely degraded 20 μM C6-HSL by 4.5 h incubation, suggesting higher AHL-degrading activities than those of other strains. In contrast, most C10-HSL remained by 4.5 h incubation in the supernatants of *S. carnosus*, *S. haemolyticus*, and *S. saprophyticus*. Lower levels of C10-HSL were detected in the culture supernatants of *S. sciuri* ATCC 29060 and 29061, which also had higher C6-HSL-degrading activities. After 9 h incubation, all CNS strains completely degraded 20 µM C10-HSL except for *S. haemolyticus* NBRC 109768. These results suggested that the CNS strains tested in this study had higher degrading activities toward C6-HSL than C10-HSL.

### 2.2. AiiA-Type AHL Lactonase Gene Is Present in the CNS Strains

**Figure 2:**
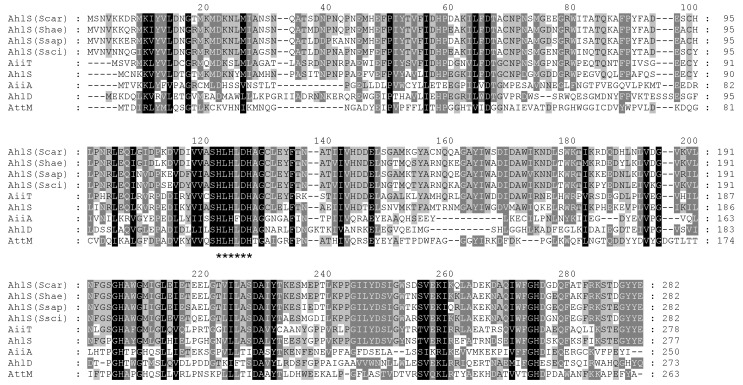
Comparisons of AhlS from the CNS strains with the known AiiA-like AHL lactonases. The amino acid sequences of AhlS from *S. carnosus* NBRC 109622 (Scar), *S. haemolyticus* NBRC 109768 (Shae), *S. saprophyticus* NBRC 102446 (Ssap), and *S. sciuri* ATCC 29060 (Ssci) were compared to those of the AhlS from *Solibacillus silvestris* StLB046 (UniProt accession No. F2F233), AiiT from *Thermaerobacter marianensis* JCM 10246 (E6SI95), AiiA from *Bacillus* sp. 240B1 (Q9L8R8), AttM from *Agrobacterium tumefaciens* C58 (Q7D3U0), and *Arthrobacter* sp. IBN110 (Q7X3T2). Sequences were aligned using ClustalW and shaded using the GeneDoc software. The consensus amino acid sequence (HXHXDH) was marked with asterisks.

The whole genome sequences of *S. carnosus* NBRC 109622, *S. haemolyticus* NBRC 109768, and *S. saprophyticus* ATCC 15305 (synonym of NBRC 102446) were deposited in DDBJ/ENA/GenBank under the accession numbers BKAO01000000, BKAY01000000, and CP035294, respectively. Gene homologs encoding the AiiA-type AHL-lactonase were searched in these genome sequences. Based on the Basic Local Alignment Search Tool (BLAST) analysis [15], the deduced amino acid sequences of SCA04_19880 from *S. carnosus* NBRC 109622, SHA04_07910 from *S. haemolyticus* NBRC 109768, and EQ030_01160 from *S. saprophyticus* NBRC 102446 showed similarity to the known AiiA-type AHL lactonases. Specifically, the amino acid sequences of these gene homologs showed higher similarity with AhlS from *S. silvestris* StLB046 [7] and AiiT from *T. marianensis* JCM 10246 [11]. Although the genome sequences of *S. sciuri* ATCC 29060 and 29061 have not been released, the *ahlS* gene homolog (FPV13_12710) was found in the genome of *S. sciuri* B9-58B (accession No. CP041879) [16]. Therefore, specific polymerase chain reaction (PCR) primers were designed based on the sequence of FPV13_12710 from *S. sciuri* B9-58B, and the *ahlS* homolog genes were successfully amplified by PCR from the genomes of ATCC 29060, ATCC 29061, and StLB252. Sequencing of the three *ahlS* genes from *S. sciuri* revealed high similarity in their amino acid sequences (over 98%). The amino acid sequences of AhlS from the CNS strains were also compared to those of the known AiiA-type AHL lactonases (Figure 2), which showed some well-conserved amino acid residues. Specifically, the zinc-binding “HXHXDH” motifs, which were commonly conserved sequences of the known AiiA-type AHL lactonases [6], were also found in the AhlS from the CNS strains (Figure 2). Interestingly, the AhlS from the CNS strains were phylogenetically related to AhlS from *S. silvestris* and AiiT from *T. marianensis* but were slightly distant from the other AiiA-type AHL lactonases (Figure S1). Therefore, these proteins might be classified as members of a new subfamily composed of AiiA-type AHL lactonases.

### 2.3. Characterization of the AHL-Degrading Activity of AhlS from the CNS Strains

**Figure 3:**
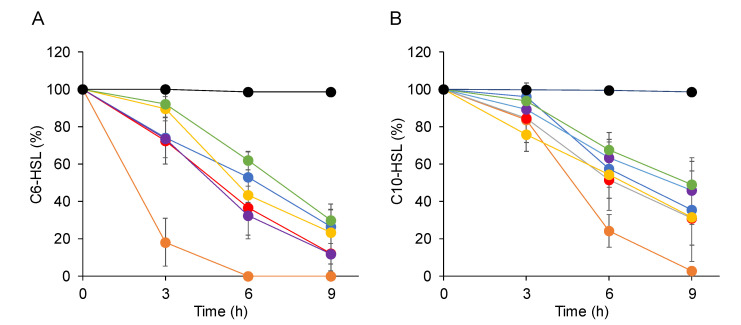
Degradation of C6-HSL (**A**) and C10-HSL (**B**) by *Escherichia coli* DH5α harboring *ahlS* genes from the CNS strains. Full-grown cultures of *E. coli* DH5α harboring an empty vector pBBR1MCS5 (black) or pBBR1MCS5 containing the *ahlS* gene from *S. carnosus* NBRC 109622 (blue), *S. haemolyticus* NBRC 109768 (orange), *S. saprophyticus* NBRC 102446 (red), *S. sciuri* ATCC 29060 (yellow), *S. sciuri* 29061 (purple), and *S. sciuri* StLB252 (green) were inoculated into the Luria–Bertani (LB) medium containing 10 μM C6-HSL or C10-HSL and incubated at 37 °C for 3, 6, and 9 h. After incubation, the remaining AHLs in the culture supernatants were visualized by *C. violaceum* and calculated using the relationship equations based on the size of the purple-color zones. The data were reproduced at least three times and the error bars indicate standard deviations.

To test if the AhlS from the CNS strains had AHL-degrading activities, the *ahlS* genes from the CNS strains were amplified by PCR and cloned into pBBR1MCS5 cloning vectors *Escherichia coli* DH5α, harboring *ahlS* gene from CNS strains, was grown in the LB medium containing 10 μM C6-HSL or C10-HSL. After 3, 6, and 9 h incubations, the remaining AHLs in the culture supernatants were visualized and quantified by the AHL reporter strains. The results indicated that over 60% of C6-HSL was degraded by 9 h incubation by *E. coli* DH5α harboring *ahlS* from the CNS strains (Figure 3A). Specifically, AhlS from *S. haemolyticus* showed the highest activity by completely degrading C6-HSL after 6 h incubation. On the other hand, *E. coli* DH5α harboring the *ahlS* from the CNS strains showed slightly lower degrading activity against C10-HSL than C6-HSL (Figure 3B). One of the AHL-degrading enzymes, AHL lactonase, degrades AHLs by hydrolyzing its homoserine lactone ring [5], which can be re-circularized in acidic solutions [17]. Therefore, AHL restoration assay was carried out to test the presence of a putative lactonase activity. The results showed that C10-HSL degraded by *E. coli* harboring *ahlS* from the CNS strains was restored by acidification (Figure S2). These results indicated that AhlS from the CNS strains worked as AHL-lactonase, similarly to other AiiA-type AHL lactonases.

### 2.4. AhlS Inhibits the Quorum-Sensing Phenotypes in Pseudomonas aeruginosa

**Figure 4:**
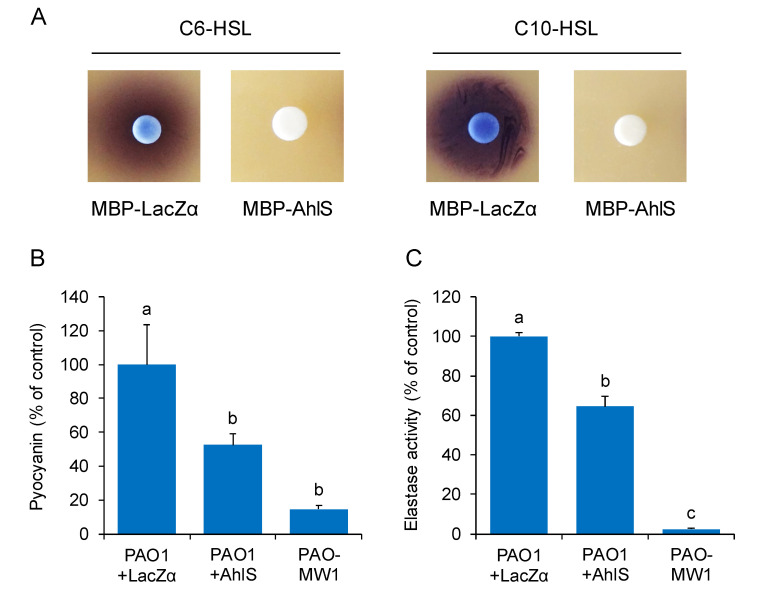
(**A**) AHL-degrading activities of MBP-LacZα and MBP-AhlS. Purified proteins were mixed with an equal volume of 20 μM C6-HSL or C10-HSL solutions and incubated at 37 °C for 30 min. The residual AHL was detected using *C. violaceum* CV026 or VIR07, respectively. Pyocyanin production (**B**) and elastase activity (**C**) in culture supernatants of *Pseudomonas aeruginosa* PAO1 treated with MBP-LacZα or MBP-AhlS. PAO-MW1 was used as the quorum-sensing-negative control. The average value of PAO1-LacZα was defined as 100%. The data were reproduced at least three times and the error bars indicate standard deviations. Different lowercase letters indicate significant differences, as determined by the Tukey’s HSD test (*p* < 0.05).

To evaluate the potential of AhlS to interfere with quorum sensing in other bacteria, AhlS was expressed and purified as a maltose-binding protein (MBP). First, the *ahlS* gene from *S. sciuri* ATCC 29060 was amplified by PCR and cloned into the pMAL-c2x vector. The MBP-AhlS fusion protein was overexpressed in *E. coli* DH5α and purified by maltose affinity chromatography. Sodium dodecyl sulfate-polyacrylamide gel electrophoresis (SDS-PAGE) analysis revealed that the overexpressed protein was approximately 75 kDa in size (Figure S3), which was congruent with the predicted molecular weight of MBP-AhlS based on its amino acid sequence. To assess the AHL-degrading activity, MBP-AhlS was mixed with 20 μM C6-HSL and C10-HSL and incubated for 30 min at 37 ℃. The results showed that MBP-AhlS completely degraded C6-HSL and C10-HSL within 30 min, whereas MBP-LacZα as a negative control did not (Figure 4A).

*P. aeruginosa* is an opportunistic pathogen that uses the quorum-sensing system to regulate several virulence factors [18]. In *P. aeruginosa* PAO1, LasI and RhlI are responsible for the synthesis of the *las* and *rhl* signals, *N*-(3-oxododecanoyl)-l-homoserine lactone (3-oxo-C12-HSL) and *N*-butyryl-l-homoserine lactone (C4-HSL), respectively [18]. Pyocyanin, which is a redox-active phenazine pigment and induces cytotoxicity in human cells [19], is produced by *P. aeruginosa* in response to AHL-mediated quorum sensing [18]. Elastase, a major virulence factor in *P. aeruginosa* that degrades tissue matrix proteins in the human host, is also regulated by the quorum-sensing system [18]. Pyocyanin production and elastase activity were drastically reduced in the *lasI rhlI* double mutant *P. aeruginosa* PAO-MW1 (Figure 4B,C). Assessment of the effect of MBP-AhlS on quorum sensing in *P. aeruginosa* showed that the pyocyanin production by *P. aeruginosa* PAO1 treated with MBP-AhlS was reduced to less than 50% of that with the control MBP-LacZα (Figure 4B). The elastase activity in *P. aeruginosa* PAO1 treated with MBP-AhlS was reduced to 60% of that with MBP-LacZα (Figure 4C). These results demonstrated that treatment by AhlS contributed to reducing the production of several virulence factors regulated by AHL-mediated quorum sensing in *P. aeruginosa*.

## 3. Materials and Methods

### 3.1. Bacterial Strains, Compounds, and Incubation Conditions

*S. carnosus* NBRC 109622^T^ [20], *S. haemolyticus* NBRC 109768^T^ [21], and *S. saprophyticus* NBRC 102446^T^ [21] were obtained from the NITE Biological Resource Center (NBRC), Japan. *S. sciuri* ATCC 29060 and 29061 [22] were obtained from the American Type Culture Collection (ATCC). *S. sciuri* StLB252 was isolated from the surface of the potato leaf in our unpublished experiment. Bacterial strains were incubated at 37 °C in the Luria–Bertani (LB) medium [23]. Solid bacterial media were made by adding agar at a final concentration of 1.5%. Ampicillin and gentamycin were added at final concentrations of 100 µg/mL and 10 µg/mL, respectively. The C6-HSL and C10-HSL used in this study were synthesized by previously-described methods [24].

### 3.2. Amplification and Sequencing of the ahlS Gene from S. sciuri

To amplify the *ahlS* gene from the genome of *S. sciuri*, specific PCR primers sciseq-F (5′-TTT TAT CGG CCT GGT AAT CCC GTT TTC GGG TCG C-3′) and sciseq-R (5′-TTC ATG CCC AAG TGC CAT AGG AGT AGG GC-3′) were designed based on the sequence of FPV13_12710 from *S. sciuri* B9-58B. PCR was performed with the KOD FX Neo system (Toyobo, Osaka, Japan) using the following cycling parameters: 98 °C for 10 s, 60 °C for 30 s, and 68 °C for 1.5 min for 30 cycles. PCR fragments were sequenced using a BigDye Terminator ver. 3.1 sequencing kit and an ABI 3500 Genetic Analyzer (Applied Biosystems, Tokyo, Japan).

### 3.3. Cloning of the ahlS Gene from the CNS Strains

The *ahlS* coding regions in the genomes of the CNS strains were amplified with the KOD FX Neo system using the following primer sets: car-F (5′-TCT GTC GAC ACC CTC AGT TGT AAT AAG CCA ATA CC-3′) and car-R (5′-TCT CTG CAG AAG TCA GAA TGA CAT AAT CCT GCT GC-3′) for *ahlS* from *S. carnosus*, hae-F (5′-TCT GTC GAC AAA GCA CAT AAA TAG AGC CTT CC) and hae-R (5′-TCT CTG CAG ACT GAT AAG TCT GAA TGG CAT ATC CC-3′) for *ahlS* from *S. haemolyticus*, sap-F (5′-TCT GTC GAC CTA AAA ATG ACA ACT TTG GAG GGC GC-3′) and sap-R (5′-TCT CTG CAG TTT ACC CAT TTC ACG CAA TAC TGC CG-3′) for *ahlS* from *S. saprophyticus*, and sci-F (5′-TCT GTC GAC TCG GTC CAT ATT CCA ACC TCA CC-3′) and sci-R (5′-TCT CTG CAG TCT GAA TGA CAT AAA CCT GCC GCT CC-3′) for *ahlS* from *S. sciuri*. The *Sal*I and *Pst*I restriction sites were underlined in the primer sequences. PCR was performed with the following cycling parameters: 98 °C for 10 s, 60 °C for 30 s, and 68 °C for 1 min for 30 cycles. The PCR products were digested by *Sal*I and *Pst*I and inserted into the same sites of a broad-host-range vector pBBR1MCS5 [25]. The prepared plasmids were transferred into *E. coli* DH5α and used for AHL-degradation assays.

### 3.4. Assessment of AHL-Degrading Activities

The full-grown cultures of CNS stains or *E. coli* DH5α harboring the AhlS-expressing plasmid were inoculated in fresh LB medium (1% inoculum) containing 100 μM isopropyl-β-d-thiogalactopyranoside (IPTG). C6-HSL or C10-HSL was added at a final concentration of 20 μM (for CNS strains) or 10 μM (for *E. coli*). After incubation at 37 °C, cells were removed by centrifugation to obtain the supernatant. The residual AHLs in the supernatant were detected using *C. violaceum* CV026 and VIR07. An overnight culture of CV026 or VIR07 was mixed with 25 mL LB agar medium and poured into Petri dishes. AHL samples (20 μL) were placed in paper discs (8 mm in diameter, Advantec, Tokyo, Japan), which were subsequently placed onto LB agar plates containing CV026 or VIR07. Assay plates were incubated overnight at 30 °C and the residual amounts of AHL were calculated using the relationship equations based on the size of the purple-colored zones and the known quantities of AHLs [26].

### 3.5. AHL Restoration Assay

The AHL-lactonase activity was assessed based on previously-established methods [27]. The full-grown culture of *E. coli* DH5α harboring the AhlS-expressing plasmid was inoculated in 4 mL fresh LB medium (1% inoculum) containing 100 μM IPTG and 10 μM C10-HSL. After incubation, the cells were removed by centrifugation at 12,000× *g* for 5 min, and 90 μL of the supernatant was mixed with 10 μL of 1 N HCl. After incubation for 48 h at 4 °C, 20 μL of 1 M phosphate buffer (pH 7) was added to neutralize the pH. The restored C10-HSL was detected with a VIR07 biosensor.

### 3.6. Purification of AhlS as an MBP Fusion

The *ahlS* gene from *S. sciuri* ATCC 29060 was amplified with the KOD FX Neo system using the following set of primers: MBP-F (5′-TCT GGA TCC ATG GTA AAT GTA AAT AAC CAA GG-3′) and MBP-R (5′-TCT CTG CAG TCT GAA TGA CAT AAA CCT GCC GCT CC-3′). The *Bam*HI and *Pst*I restriction sites were underlined in the primer sequences. PCR was performed with the following cycling parameters: 98 °C for 10 s, 60 °C for 30 s, and 68 °C for 1 min for 30 cycles. The PCR products were digested by *Bam*HI and *Pst*I and inserted into the same sites of a pMAL-c2X vector (New England Biolabs, Tokyo, Japan) to construct pMAL-ahlS.

The full-grown culture of *E. coli* DH5α harboring pMAL-ahlS was inoculated into 100 mL fresh LB medium containing ampicillin. After incubation at 37 °C for 2 h, the expression of MBP-AhlS was induced by adding 1 mM IPTG. After incubation for 18 h at 37 °C, cells were harvested by centrifugation and resuspended in the column buffer (20 mM Tris-HCl buffer and 200 mM NaCl, pH 7.4). Lysozyme from egg white (Wako, Osaka, Japan) was added to the suspension at a final concentration of 250 μg/mL. After incubation for 4 min at 37 °C, the suspension was sonicated and centrifuged to remove cell debris. The filtrated sample was loaded on an MBPTrap affinity chromatography column (GE Healthcare, Tokyo, Japan) equilibrated with the column buffer and eluted with 10 mM maltose. As the negative control, MBP-LacZα fusion was expressed and purified from cells of *E. coli* DH5α harboring pMAL-c2X using the same method. The expression and purification of MBP fusion proteins were analyzed on 10% SDS-PAGE followed by Coomassie Brilliant Blue staining.

### 3.7. Inhibition of Quorum Sensing by MBP-AhlS in P. aeruginosa

*P. aeruginosa* PAO1 [28] and its quorum-sensing-deficient mutant PAO-MW1 [29] were incubated overnight at 37 °C in the LB medium, and 40 μL of each was inoculated into 4 mL PTSB medium [30] for the pyocyanin assay or 4 mL LB medium for the elastase assay. The 200 μL of AhlS solution (approximately 40 μg/μL) or column buffer (200 μL, the negative control) was added and incubated for 17 h at 37 °C. For the pyocyanin production assay, 450 μL chloroform was mixed with 750 μL of each culture supernatant and vortexed for 10 s. The chloroform layer was transferred to a fresh tube, mixed with 150 μL 0.2 M HCl, and vortexed for 10 s. After centrifugation, the absorbance of the aqueous layer was measured at 520 nm. For the elastase assay, 10 mg elastin–Congo red was added to 100 μL of each culture supernatant, which was then mixed with 900 μL of the assay buffer (0.1 M Tris-HCl, 1 mM CaCl_2_, pH 8.0). After incubation for 4 h at 37 °C with shaking at 1400 rpm, the precipitate was removed by centrifugation, and the absorbance was measured at 495 nm. The relative pyocyanin production (A_520_/OD_600_) and elastase activity (A_495_/OD_600_) of PAO1-AhlS were calculated with those of PAO1- LacZα set to 100%.

### 3.8. Nucleotide Sequence Accession Numbers

The nucleotide sequences of *ahlS* from *S. sciuri* ATCC 29060, 29061, and StLB252 have been deposited in the DDBJ/ENA/GenBank databases under the following accession number LC128625, LC128626, and LC128627, respectively.

## 4. Conclusions

In summary, our work is the first to report that *ahlS* from the CNS strains has AHL-degrading activity. It has been reported that CNS species are part of the normal flora on the skin and are less virulent. One of the CNS species, *Staphylococcus epidermidis,* is a normal colonizer of the skin and can be isolated from the diseased tissue alongside *P. aeruginosa* [31]. Interestingly, the extracellular substance secreted by *P. aeruginosa* affected the biofilm formation of *S. epidermidis* [31,32]. Since *P. aeruginosa* uses quorum sensing to regulate biofilm formation [33], it was assumed that the inhibition of quorum sensing by AhlS from the CNS strains was related to the niche competition against *P. aeruginosa*. Based on the results from the BLAST search, AhlS was also present in the genome of *S. epidermidis* NCTC 4133 (accession No. LR134242). Therefore, *ahlS* genes are assumed to be widely distributed among CNS strains. In this study, we used the purified AhlS protein for the quorum-quenching assay and demonstrated the inhibition of virulence factors, which are regulated by quorum sensing in *P. aeruginosa*. CNS strains might contribute to the inhibition of the quorum-sensing system of other pathogens in the normal flora of the skin. Meanwhile, it has not been elucidated whether CNS strains produce sufficient levels of AhlS for inhibition of quorum-sensing in other bacteria in natural systems, and the cell numbers of CNS strains are abundant enough within the community to impact the quorum-sensing system of more abundant microbes. Future studies could investigate how the behavior of AHL production and degradation on the surface of skins might contribute to elucidating the complicated quorum-sensing networks in the normal flora of the skin.

## Supplementary Materials

The following are available online at https://www.mdpi.com/2079-6382/9/8/483/s1, Figure S1: Phylogenetic tree based on amino acid sequences of AhlS from CNS strains, Figure S2: Restoration of C10-HSL degraded by *E. coli* DH5α harboring *ahlS* from CNS strains, Figure S3: SDS-PAGE analysis of MBP-AhlS.

## References

[1] Waters C.M., Bassler B.L. (2005). Quorum sensing: Cell-to-cell communication in bacteria. Annu. Rev. Cell. Dev. Biol..

[2] Parsek M.R., Greenberg E.P. (2000). Acyl-homoserine lactone quorum sensing in gram-negative bacteria: A signaling mechanism involved in associations with higher organisms. Proc. Nat. Acad. Sci. USA.

[3] De Kievit T.R., Iglewski B.H. (2000). Bacterial quorum sensing in pathogenic relationships. Infect. Immun..

[4] Von Bodman S.B., Bauer W.D., Coplin D.L. (2003). Quorum sensing in plant-pathogenic bacteria. Annu. Rev. Phytopathol..

[5] Uroz S., Dessaux Y., Oger P. (2009). Quorum sensing and quorum quenching: The yin and yang of bacterial communication. ChemBioChem.

[6] Dong Y.H., Xu J.L., Li X.Z., Zhang L.H. (2000). AiiA, an enzyme that inactivates the acylhomoserine lactone quorum-sensing signal and attenuates the virulence of *Erwinia carotovora*. Proc. Nat. Acad. Sci. USA.

[7] Morohoshi T., Tominaga Y., Someya N., Ikeda T. (2012). Complete genome sequence and characterization of the *N*-acylhomoserine lactone-degrading gene of the potato leaf-associated *Solibacillus silvestris*. J. Biosci. Bioeng..

[8] Zhang H.B., Wang L.H., Zhang L.H. (2002). Genetic control of quorum-sensing signal turnover in *Agrobacterium tumefaciens*. Proc. Nat. Acad. Sci. USA.

[9] Park S.Y., Lee S.J., Oh T.K., Oh J.W., Koo B.T., Yum D.Y., Lee J.K. (2003). AhlD, an *N*-acylhomoserine lactonase in *Arthrobacter* sp., and predicted homologues in other bacteria. Microbiology.

[10] Wang W.Z., Morohoshi T., Someya N., Ikeda T. (2012). AidC, a novel *N*-acylhomoserine lactonase from the potato root-associated *Cytophaga-Flavobacteria-Bacteroides* (CFB) group bacterium *Chryseobacterium* sp. strain StRB126. Appl. Environ. Microbiol..

[11] Morohoshi T., Tominaga Y., Someya N., Ikeda T. (2015). Characterization of a novel thermostable *N*-acylhomoserine lactonase from the thermophilic bacterium *Thermaerobacter marianensis*. J. Biosci. Bioeng..

[12] Huebner J., Goldmann D.A. (1999). Coagulase-negative staphylococci: Role as pathogens. Annu. Rev. Med..

[13] McClean K.H., Winson M.K., Fish L., Taylor A., Chhabra S.R., Camara M., Daykin M., Lamb J.H., Swift S., Bycroft B.W. (1997). Quorum sensing and *Chromobacterium violaceum*: Exploitation of violacein production and inhibition for the detection of *N*-acylhomoserine lactones. Microbiology.

[14] Morohoshi T., Kato M., Fukamachi K., Kato N., Ikeda T. (2008). *N*-acylhomoserine lactone regulates violacein production in *Chromobacterium violaceum* type strain ATCC 12472. FEMS Microbiol. Lett..

[15] Altschul S.F., Gish W., Miller W., Myers E.W., Lipman D.J. (1990). Basic local alignment search tool. J. Mol. Biol..

[16] Neyaz L., Karki A.B., Fakhr M.K. (2020). Draft genome sequence of megaplasmid-bearing *Staphylococcus sciuri* strain B9-58B, isolated from retail pork. Microbiol. Resour. Announc..

[17] Morohoshi T., Someya N., Ikeda T. (2009). Novel *N*-acylhomoserine lactone-degrading bacteria isolated from the leaf surface of *Solanum tuberosum* and their quorum-quenching properties. Biosci. Biotechnol. Biochem..

[18] Rumbaugh K.P., Griswold J.A., Iglewski B.H., Hamood A.N. (1999). Contribution of quorum sensing to the virulence of *Pseudomonas aeruginosa* in vurn wound infections. Infect. Immun..

[19] Priyaja P., Jayesh P., Philip R., Bright Singh I.S. (2016). Pyocyanin induced in vitro oxidative damage and its toxicity level in human, fish and insect cell lines for its selective biological applications. Cytotechnology.

[20] Schleifer K.H., Fischer U. (1982). Description of a new species of the genus *Staphylococcus*: *Staphylococcus carnosus*. Int. J. Syst. Evol. Microbiol..

[21] Schleifer K.H., Kloos W.E. (1975). Isolation and characterization of *Staphylococci* from human skin I. amended descriptions of *Staphylococcus epidermidis* and *Staphylococcus saprophyticus* and descriptions of three new species: *Staphylococcus cohnii*, *Staphylococcus haemolyticus* and *Staphylococcus xylosus*. Int. J. Syst. Evol. Microbiol..

[22] Kloos W.E., Schleifer K.H., Smith R.F. (1976). Characterization of *Staphylococcus sciuri* sp. nov. and its subspecies. Int. J. Syst. Evol. Microbiol..

[23] Sambrook J. (2001). Molecular Cloning: A Laboratory Manual/Joseph Sambrook, David, W. Russell.

[24] Chhabra S.R., Harty C., Hooi D.S., Daykin M., Williams P., Telford G., Pritchard D.I., Bycroft B.W. (2003). Synthetic analogues of the bacterial signal (quorum sensing) molecule *N*-(3-oxododecanoyl)-l-homoserine lactone as immune modulators. J. Med. Chem..

[25] Kovach M.E., Elzer P.H., Hill D.S., Robertson G.T., Farris M.A., Roop R.M., Peterson K.M. (1995). Four new derivatives of the broad-host-range cloning vector pBBR1MCS, carrying different antibiotic-resistance cassettes. Gene.

[26] Ochiai S., Yasumoto S., Morohoshi T., Ikeda T. (2014). AmiE, a novel *N*-acylhomoserine lactone acylase belonging to the amidase family, from the activated-sludge isolate *Acinetobacter* sp. strain Ooi24. Appl. Environ. Microbiol..

[27] Ochiai S., Morohoshi T., Kurabeishi A., Shinozaki M., Fujita H., Sawada I., Ikeda T. (2013). Production and degradation of *N*-acylhomoserine lactone quorum sensing signal molecules in bacteria isolated from activated sludge. Biosci. Biotechnol. Biochem..

[28] Holloway B.W., Krishnapillai V., Morgan A.F. (1979). Chromosomal genetics of *Pseudomonas*. Microbiol. Rev..

[29] Whiteley M., Lee K.M., Greenberg E.P. (1999). Identification of genes controlled by quorum sensing in *Pseudomonas aeruginosa*. Proc. Nat. Acad. Sci. USA.

[30] Ishida T., Ikeda T., Takiguchi N., Kuroda A., Ohtake H., Kato J. (2007). Inhibition of quorum sensing in *Pseudomonas aeruginosa* by *N*-acyl cyclopentylamides. Appl. Environ. Microbiol..

[31] Pihl M., Chávez de Paz L.E., Schmidtchen A., Svensäter G., Davies J.R. (2010). Effects of clinical isolates of *Pseudomonas aeruginosa* on *Staphylococcus epidermidis* biofilm formation. FEMS Immunol. Med. Microbiol..

[32] Pihl M., Arvidsson A., Skepö M., Nilsson M., Givskov M., Tolker-Nielsen T., Svensäter G., Davies J.R. (2013). Biofilm formation by *Staphylococcus epidermidis* on peritoneal dialysis catheters and the effects of extracellular products from *Pseudomonas aeruginosa*. Pathog. Dis..

[33] de Kievit T.R. (2009). Quorum sensing in *Pseudomonas aeruginosa* biofilms. Environ. Microbiol..

