# Supplementary material for: Identification and Characterization of Quorum-Quenching Activity of N-Acylhomoserine Lactonase from Coagulase-Negative Staphylococci
Source: Antibiotics (Basel). 2020 Aug 5;9(8):483. doi: 10.3390/antibiotics9080483 (PMC7459623; doi:10.3390/antibiotics9080483)
Supplement: Supplementary file 1 [file antibiotics-09-00483-s001.pdf]

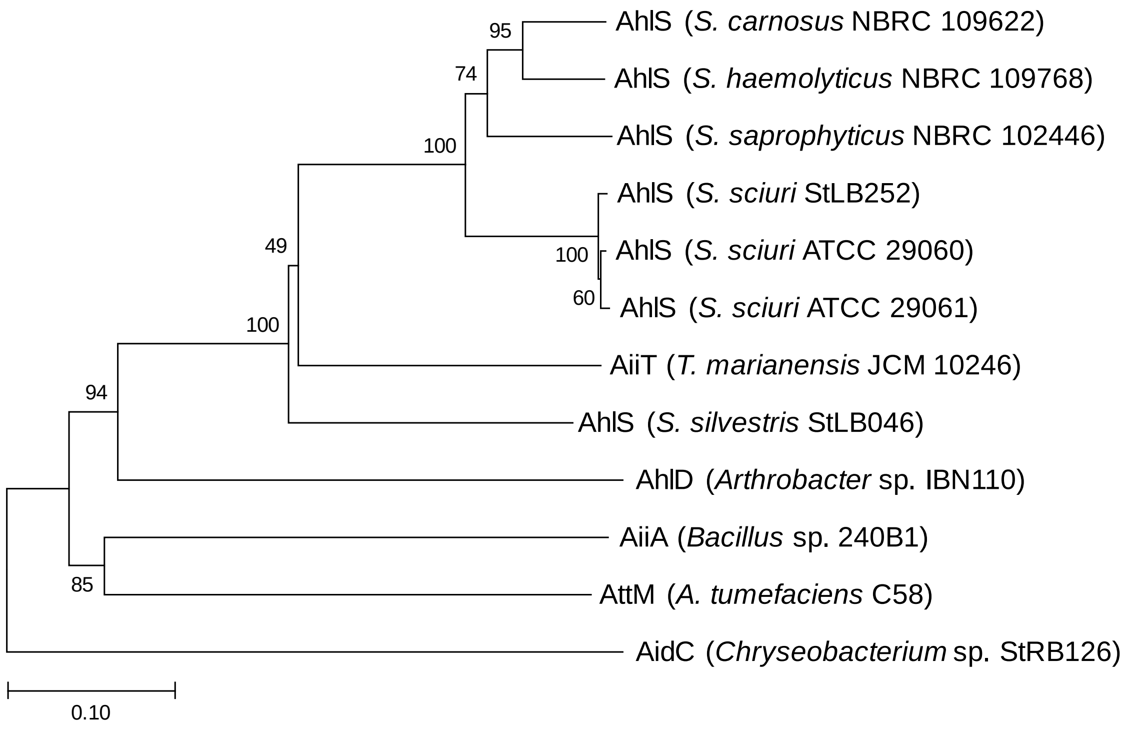

Figure S1. Phylogenetic tree based on amino acid sequences of AhlS from CNS strains, AhlS from *S. silvestris* StLB046 (UniProt accession no. F2F233), AiiT from *T. marianensis* JCM 10246 (E6SI95), AiiA from *Bacillus* sp. 240B1 (Q9L8R8), AttM from *A. tumefaciens* C58 (Q7D3U0), *Arthrobacter* sp. IBN110 (Q7X3T2), and AidC from *Chryseobacterium* sp. StRB126 (I7HB71). The phylogenetic tree was constructed by the neighbor-joining method with the ClustalW program of MEGA7 (Reference 1). The percentage of replicate trees in which the associated taxa clustered together in the bootstrap test (1000 replicates) are shown next to the branches. The scale bar represents 0.1 substitutions per amino acid position.

Reference 1

Kumar, S.; Stecher, G.; Tamura, K. MEGA7: molecular evolutionary genetics analysis version 7.0 for bigger datasets. *Mol. Biol. Evol.* **2016**, 33, 1870-1874.

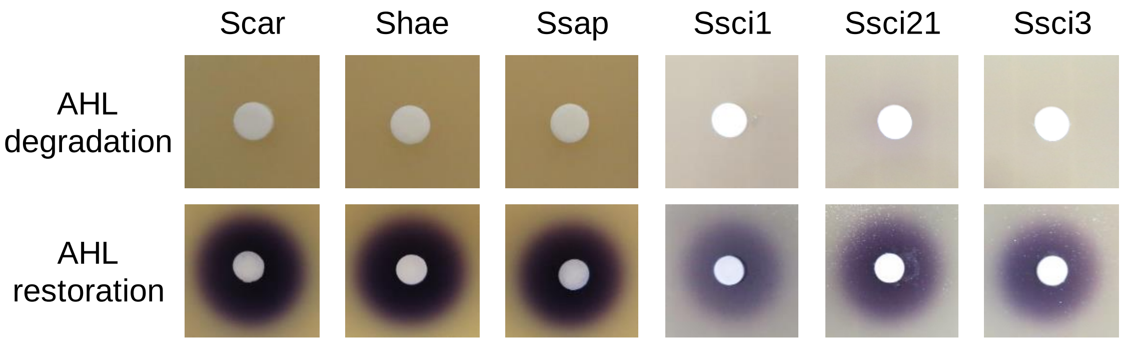

Figure S2. Restoration of C10-HSL degraded by *E. coli* DH5α harboring *ahlS* from *S. carnosus* NBRC 109622 (Scar), *S. haemolyticus* NBRC 109768 (Shae), *S. saprophyticus* NBRC 102446 (Ssap), *S. sciuri* ATCC 29060 (Ssci1), *S. sciuri* 29061 (Ssci2), and *S. sciuri* StLB252 (Ssci3). *E. coli* DH5α harboring the AhlS-expressing plasmid was incubated for 20 h in LB medium containing 20 μM C10-HSL. The degraded C10-HSL was re-circularized by acidification. The remaining and restored C10-HSL was visualized with the VIR07 reporter strain.

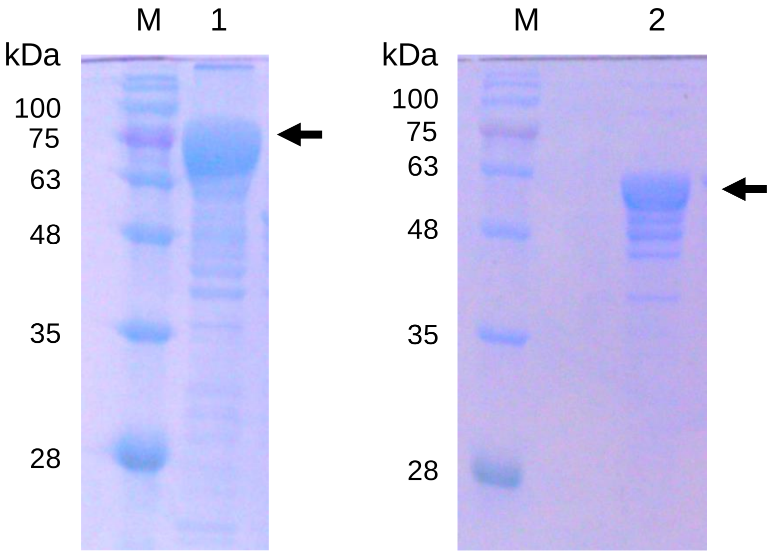

Figure S3. Purification and identification of MBP-AhlS and MBP-LacZα. Lane M, protein molecular weight marker (Nihon Genetics, Tokyo, Japan); lane 1, purified MBP-AhlS (approximately 75 kDa); and lane 2, purified MBP-LacZα (approximately 51 kDa). Samples were analyzed by SDS-PAGE in a 10% polyacrylamide gel. The protein band of MBP-AhlS and MBP-LacZα was indicated by an arrow.
